# Deciphering the dark proteome of Chikungunya virus

**DOI:** 10.1038/s41598-018-23969-0

**Published:** 2018-04-11

**Authors:** Ankur Singh, Ankur Kumar, Rakhi Yadav, Vladimir N. Uversky, Rajanish Giri

**Affiliations:** 1 School of Basic Sciences, Indian Institute of Technology Mandi, Himachal Pradesh, 175005 India; 2 Department of Molecular Medicine and Byrd Alzheimer’s Research Institute, Morsani College of Medicine, University of South Florida, Tampa, Florida United States of America; 3 Laboratory of New Methods in Biology, Institute for Biological Instrumentation, Russian Academy of Sciences, Pushchino, Moscow Region Russia; 4 BioX Centre, Indian Institute of Technology Mandi, VPO Kamand, 175005 India

## Abstract

Chikungunya virus (CHIKV) is a mosquito-borne alphavirus. The outbreak of CHIKV infection has been seen in many tropical and subtropical regions of the biosphere. Current reports evidenced that after outbreaks in 2005–06, the fitness of this virus propagating in *Aedes albopictus* enhanced due to the epistatic mutational changes in its envelope protein. In our study, we evaluated the prevalence of intrinsically disordered proteins (IDPs) and IDP regions (IDPRs) in CHIKV proteome. IDPs/IDPRs are known as members of a ‘Dark Proteome’ that defined as a set of polypeptide segments or whole protein without unique three-dimensional structure within the cellular milieu but with significant biological functions, such as cell cycle regulation, control of signaling pathways, and maintenance of viral proteomes. However, the intrinsically disordered aspects of CHIKV proteome and roles of IDPs/IDPRs in the pathogenic mechanism of this important virus have not been evaluated as of yet. There are no existing reports on the analysis of intrinsic disorder status of CHIKV. To fulfil this goal, we have analyzed the abundance and functionality of IDPs/IDPRs in CHIKV proteins, involved in the replication and maturation. It is likely that these IDPs/IDPRs can serve as novel targets for disorder based drug design.

## Introduction

Chikungunya fever is triggered by an arthropod-borne virus (arbovirus) known as Chikungunya virus (CHIKV) that is transmitted by mosquitoes (*Aedes aegypti* and *Aedes albopictus*)^1^ and is disseminated at a higher rate in tropical regions. A Makonde word ‘Chikungunya’, which means: ‘The one which bends up’^2^, is taken from the Bantu language. The first epidemic of this disease was recognized in Tanzania in 1952. Since then, it had been considered as a tropical neglected disease^3^, despite the fact that in past 50 years, innumerable cases of re-emergence of CHIKV have been documented in the African and Asian continents^4^. However, after its recent outbreaks and the disease severity, it is listed now as a category C priority pathogen in US National Institute of Allergy and Infectious Diseases (NIAID)^5^. The main manifestations are flu-like symptoms, such as fever, headache, joint pain, and difficulties in movement^2^. The CHIKV infection has a rapid onset and it gets cleared in 5–7 days^6^. The fitness of this virus propagating in *Aedes albopictus* is enhanced due to the epistatic mutational changes in its envelope protein^7,8^. Dong *et al*., also explained the infection pattern of CHIKV in midguts and salivary glands of two different strains of *Aedes aegypti*, Higgs white eyes (HWE) and Orlando, Florida (ORL)^9^. Mutational pressure is generally high in the viral genome, and similar patterns are observed in CHIKV and Zika virus^7,10^.

**Figure 1:**
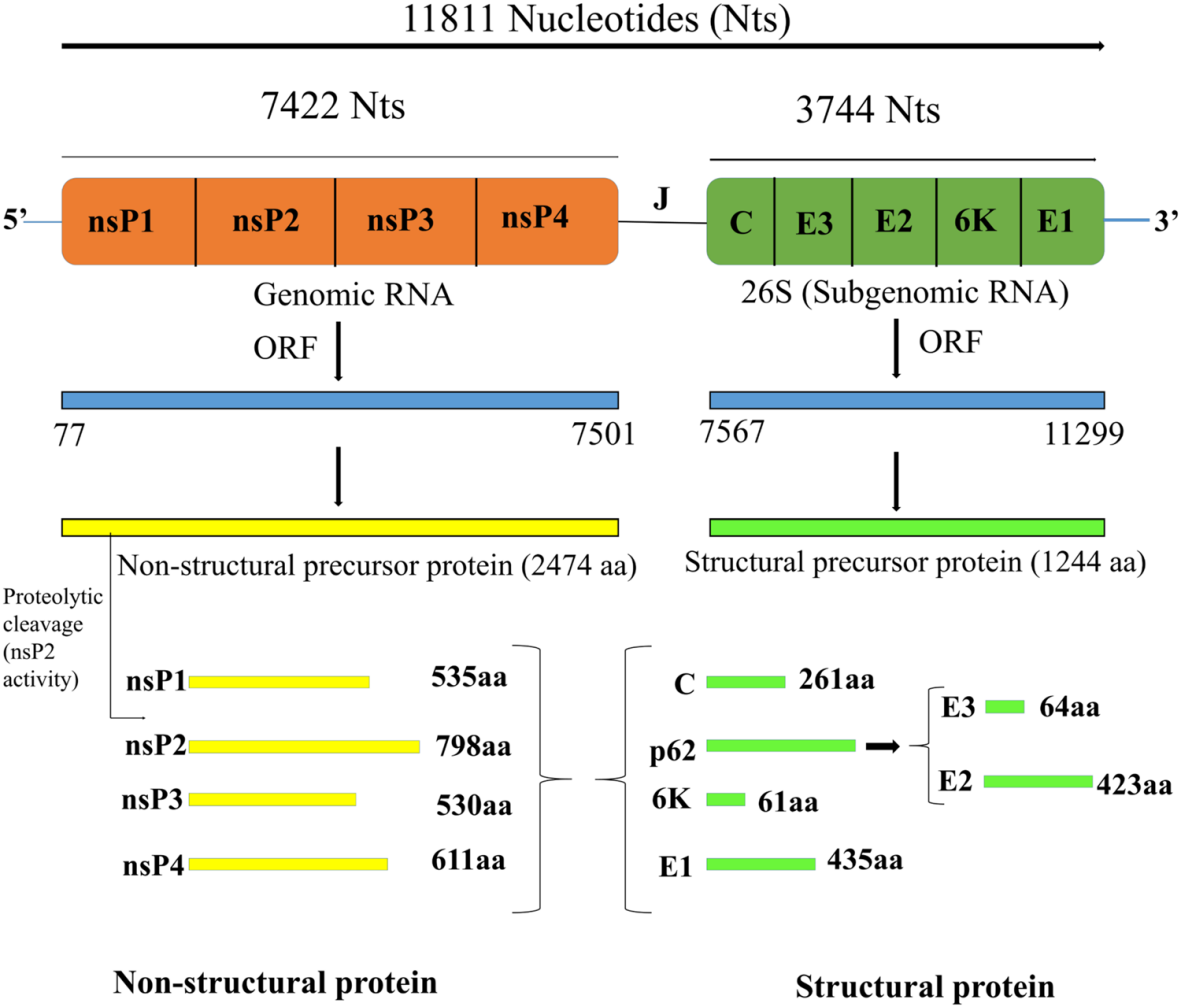
Confinement of all proteins within CHIKV genome (Q8JUX6 and Q8JUX5). The CHIKV RNA (11811 nucleotides) translates into non-structural precursor proteins of 2474 residues and structural precursor protein of 1244 residues. On maturation, non-structural precursor protein cleaved into nsP1, nsP2, nsP3 and nsP4, and structural precursor protein cleaved into C, E1, E2, E3 and 6K proteins.

CHIKV is a small spherical (diameter of about 60–70 nm) shaped virus having a single-stranded positive-sense RNA genome (11,811 nucleotides)^11^ organises as 5′UTR-nsP1-nsP2-nsP3-nsP4-J-C-E3-E2-6K-E1-polyA-3′UTR^12^. It has two open reading frames (ORFs) placed between the 5′and 3′ UTRs. First ORF (7,422 nucleotides long) encodes four non-structural proteins, nsP1 (535 residues, involved in capping and GTPase activity), nsP2 (798 residues, shows 5′ RTPase, helicase and protease activity), nsP3 (530 residues, has replicase activity and involved in RNA synthesis), and nsP4 (611 residues, has RNA-dependent RNA polymerase activity)^13^ (Fig. 1). Second ORF (3,744 nucleotides long) encodes five structural proteins, such as capsid (261 residues, involved in growth and assembly) and envelope glycoproteins E1 (439 residues, facilitate membrane fusion), E2 (423 residues, helps in receptor binding), E3 (64 residues, protects the E2-E1 heterodimer from premature fusion with cellular membrane), and J (junction) region (used as the promoter for subgenomic RNA synthesis)^13^. Recently, the structure of CHIKV was determined by the cryo-electron microscopy (PDB ID: 3J2W)^14^.

The prime focus of our article is to analyse the dark proteome of CHIKV. The dark proteome defined as proteins not amenable to structure determination by conventional methods, such as x-ray crystallography and electron microscopy^15^. Most of the dark proteome are IDPs of hybrid proteins containing ordered domains and IDPRs^16^, which show specific functions without being folded into unique 3D structure under physiological conditions^17^. It is known, hydrophobic interaction plays a driving force in protein folding^18^. Since IDPs/IDPRs are more hydrophilic than ordered proteins^19^, this could be one of the reasons for their disorderness^20^. These proteins exist as highly dynamic conformational ensembles and can attain highly diverse conformations, such as random coils, molten globules, pre-molten globules, flexible linkers, and much more^21–25^. A globular structure attained by the ordered proteins that typically prevent the formation of aggregate or misfolding because of the frustrations in amino acid sequence compositions^26^. IDPs/IDPRs are promiscuous binders that can be involved in numerous interactions with many partners, thereby acting as an important hub in protein-protein interaction networks to regulate multiple signaling pathways at a time^27–29^. Many IDPs/IDPRs show disorder to order transition after binding to their partners^30–32^. For example, transactivation domain of c-Myb is intrinsically disordered in its unbound form, but after interaction with KIX, this domain gains an α-helical conformation^33^. Some IDPs/IDPRs facilitating the interaction with partners are known as molecular recognition features (MoRFs). Most of the viral disordered proteins show MoRF regions. For example, structural and non-structural proteins of Zika virus has MoRF regions that regulate the functionality of this virus^34^.

Being disordered, these proteins increase the probability of binding to their partners by providing greater capture radii; i.e., utilizing a binding mode known as a fly-casting mechanism^35^. Despite this fly-casting mechanism, it was proposed that some disordered systems such as transactivation domain of c-Myb region, cannot speed up the recognition events between an IDP and its partner^36^. In recent studies, it is evidenced IDPs/IDPRs play a vital role in the establishment of several macromolecular complexes^37^ and also serve as assembly hubs^38^. Furthermore, it is recognized now that disordered regions represent new and attractive targets for drug design^39–42^. For example, IDPRs in c-Myc are the new targets for the development of drugs to cure cancer^43,44^. In our work, we have analyzed the dark proteome of CHIKV and evaluated disordered regions of viral proteins in terms of their functional significance.

## Results and Discussion

### Intrinsically disordered regions in CHIKV polyprotein

X-ray crystallography represents a unique technique for the determination of the 3D structure of proteins. It is based on the analysis of the scattering patterns of X-rays, which reads electron density maps to understand protein 3D structure^45^. One should keep in mind though, that the crystallization process typically requires various additives, such as PEG, high salt concentration, and many more, that might affect protein structure. IDPs/IDPRs are highly flexible and therefore characterized by the lack of specific electron densities. Since crystal structure of most of the CHIKV proteins are not reported at Protein Data Bank (PDB), we used limited crystallographic structural information that is currently available for a macro domain of nsP3 (PDB ID: 3GPG)^46^, nsP2 protease domain (PDB ID: 3TRK), and an envelope glycoprotein complex (E1, E2 and E3; PDB ID: 3N40)^47^. In light of the limited structural information, the use of computational analysis to observe disordered regions of the query protein may deliver great advantages^23^.

#### Analysis of disordered regions in non-structural proteins

**Figure 2:**
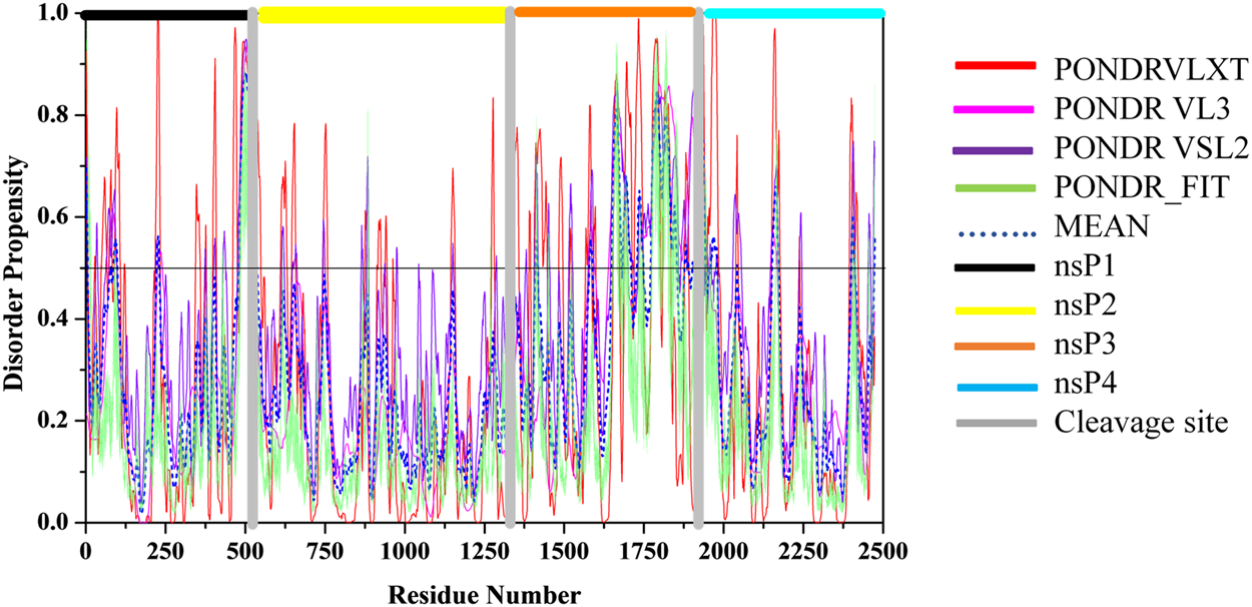
Intrinsically disordered cleavage sites in CHIKV non-structural polyprotein (Q8JUX6). Prediction of intrinsically disordered regions of non-structural CHIKV polyprotein by PONDR^®^ VSL2 (magenta line), PONDR^®^ VL3 (violet line), PONDR^®^ VLXT (red color), and PONDR-FIT (olive line). Mean disorder predisposition is shown by a dashed blue line. Localization of individual protein is represented by horizontal color bars at the top: nsP1 (black), nsP2 (yellow), nsP3 (orange), and nsP4 (light blue). Grey color vertical bar shows three cleavage sites. During the course of maturation, polyprotein cleaves into individual protein by nsP2 protease. These three cleavage sites (shown by grey vertical bars) lies at 535–536 (1^st^), 1333–1334 (2^nd^), and 1863–1864 (3^rd^) amino acid residues that show disorder regions. The amino acids and regions that have PONDR score ≥ 0.5 are considered as intrinsically disordered.

**Figure 3:**
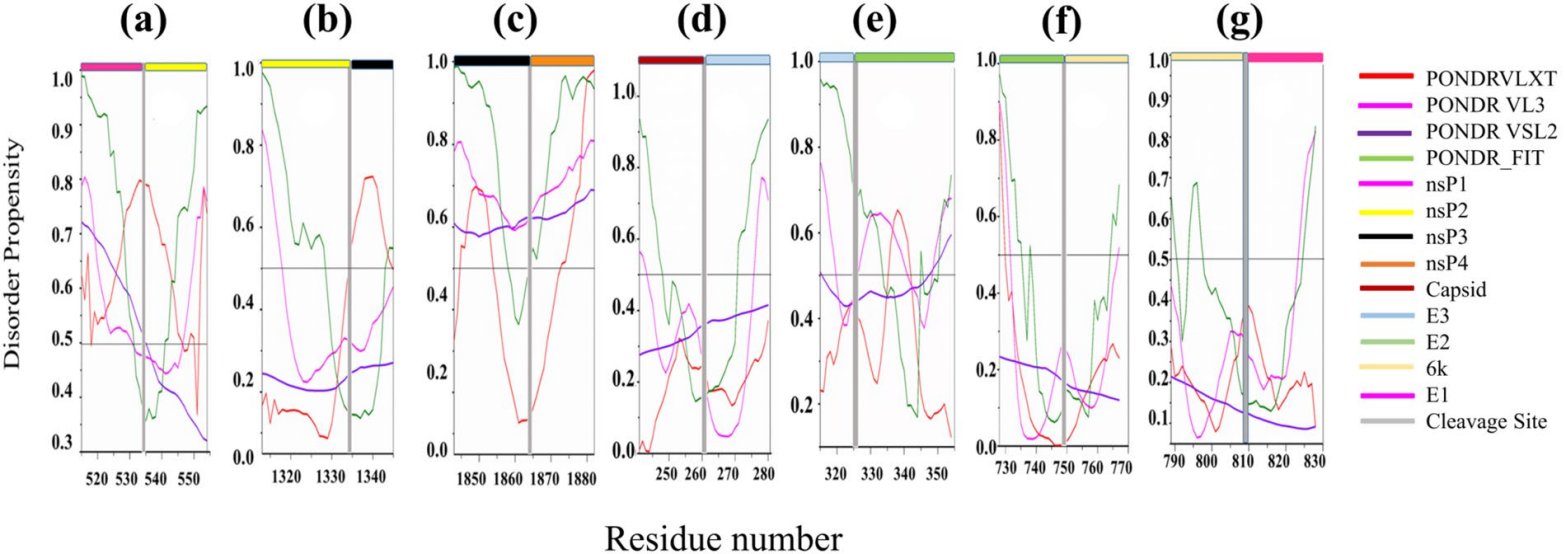
Contribution of intrinsically disordered regions in the maturation of specific proteins of CHIKV virus. The plots a-g represent the cleavage sites with respect to disordered regions present at the cleavage junction (grey vertical bar) of both CHIKV precursor proteins (cleavage of non-structural precursor protein by the nsP2 protease)^96^. Plot shows the cleavage site between (**a**) nsP1 (pink horizontal bar) and nsP2 (yellow horizontal bar) protein, (**b**) nsP2 (yellow horizontal bar) and nsP3 (black horizontal bar) protein, (**c**) nsP3 (black horizontal bar) and nsP4 (orange horizontal bar), (**d**) capsid (dark red horizontal bar) and E3 (cyan horizontal bar) protein, cleaved by capsid protease^97^, (**e**) E3 (cyan horizontal bar) and E2 (green horizontal bar) proteins, cleaved by furin cleavage, (**f**) E2 (green horizontal bar) and 6K (yellow horizontal bar) protein, cleaved by host signal peptidase, (**g**) 6K (yellow horizontal bar) and E1 (pink horizontal bar) protein, cleaved by host signal peptidase.

Non-structural precursor polyprotein P1234. Genomic RNA of alphavirus translates into a short-lived non-structural precursor polyprotein P1234 that helps in the replication process. It is synthesized because of a fraction of ribosome unable to terminate the translation when reaching the opal stop codon^48^. There are three cleavage sites lie between the residue 535–536, 1333–1334, and 1863–1864 in the P1234 (Fig. 2). These three cleavage sites are also represented with respect to disordered regions predicted at the cleavage junction in Fig. 3a–c. At the initial stage of infection, P1234 is cleaved in *trans* by the nsP2 protease, forming P123 and nsP4 protein. Then, the P123 complex and nsP4 start replicating the viral genome into antigenome. Further, P123 is cleaved in *cis* into nsP1 and P23 by the nsP2 protease. At the N-terminus of P23, an ‘activator’ is exposed and induces the cleavage of P23 into nsP2 and nsP1^49^. At the later stage of infection, P1234 is quickly cleaved by nsP2 protease into P12 and P34 and then into nsP1, nsP2, nsP3 and nsP4^49^. We will discuss the results of disorder analysis of each protein in subsequent paragraphs.

**Figure 4:**
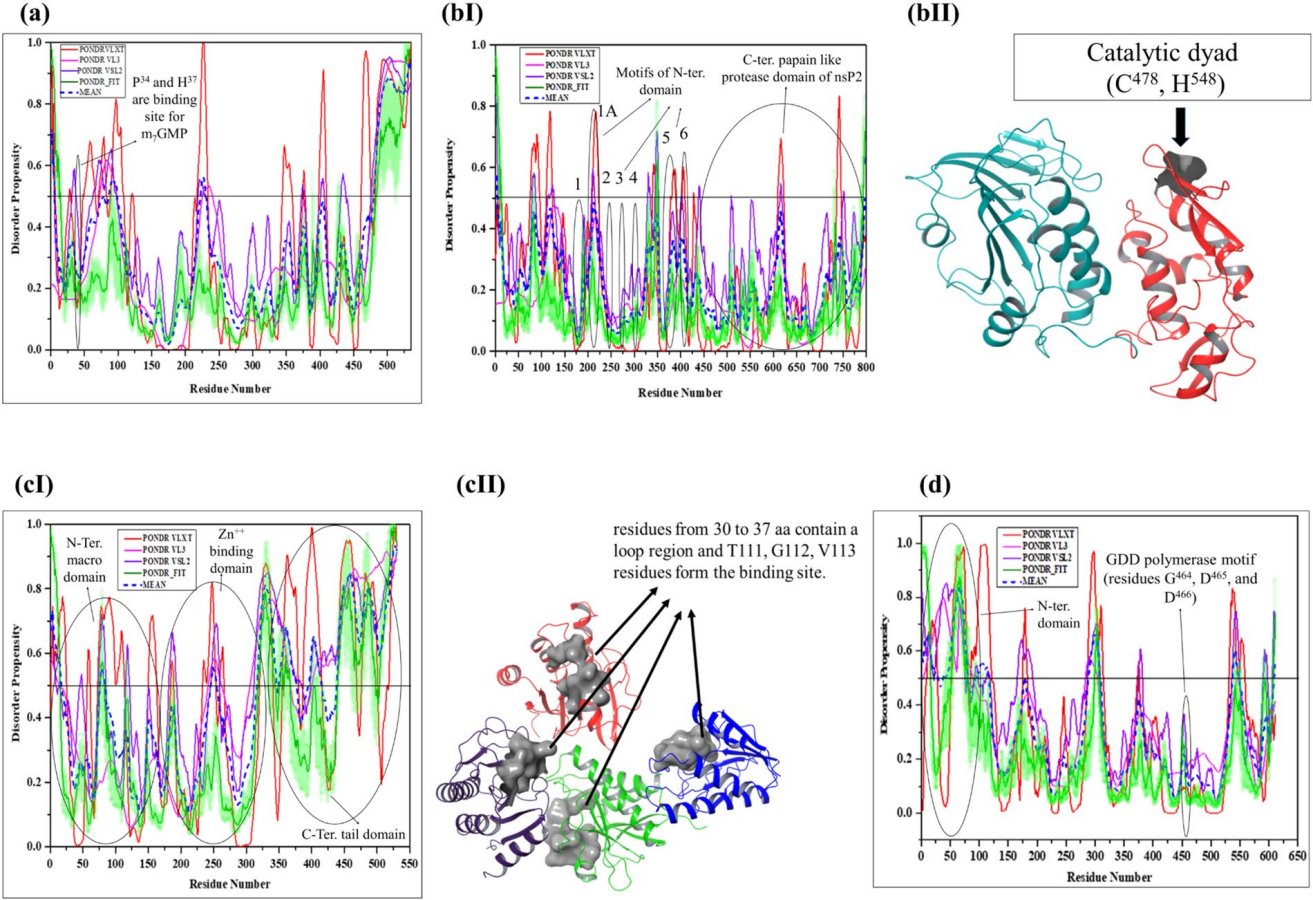
Diagrammatic representation of disordered aspect of CHIKV non-structural proteins. PONDR^R^ VSL2, VL3, VL-XT and PONDR_FIT predictors used for disorder analysis. Plots represent disorder analysis of (**a**) nsP1 (residues 1–535, denoted as 1–535 at x-axis), shows disorder regions at its C-terminal region (Mean disorder propensity ≥ 0.5, from 394^th^ to 535^th^ residues) and black encircle represents binding site for m^7^GMP (**bI**) nsP2 (residues 536–1333, denoted as 1–798 at x-axis), black encircles represent domains (**bII**) the crystal structure of nsP2 protease where C^1013^ and H^1083^ serve as catalytic dyad, are represented as C^478^ and H^548^ respectively in this figure (**cI**) nsP3 (residues 1334–1863, denoted as 1–530 at x-axis), black encircles represent domains of nsP3 (**cII**) crystal structure of macro domain of nsP3 protein (**d**) nsP4 (residues 1864–2474, denoted as 1–611 at x-axis), shows the disorder region at its N-terminal domain, black encircle represents domain.

**Figure 5:**
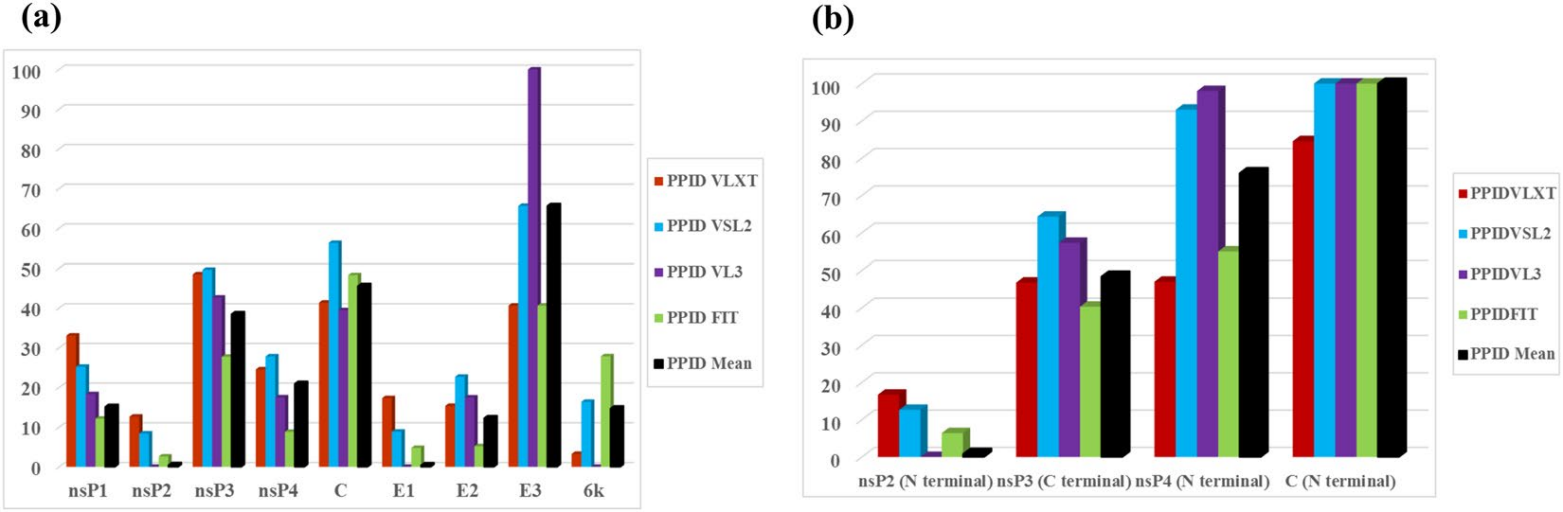
Results of predicted percentage of intrinsic disorder (PPID) in CHIKV proteins. PPID value predicted for various proteins of CHIKV is indicated at y-axis. Four softwares were used to predict the percentage disorder in proteins by VLXT, VSL2, VL3 and PONDER_FIT indicated by red, blue, violet and green colour bar. The mean PPID value of the results of these predictors is represented by a black bar. Plot (**a**) shows the disorder prediction for all CHIKV proteins and (**b**) represents disorder prediction for specific domains of non-structural and capsid proteins.

nsP1 protein. The nsP1 protein contains the methyltransferase (MTase) and guanylyltransferase (GTase) domains that have the role in 5′ capping, attachment of replication complex to the cytosolic membrane, induction of tapered pseudopodium-like structure, and in the synthesis of subgenomic RNA^50^. In the capping mechanism, methyl group from S-adenosylmethionine is transferred at the 7^th^ position of GTP by the nsP1 enzyme, which forms a covalent complex with the m^7^GMP and releases pyrophosphate. The methylated residue is transferred to 5′ end of viral RNA to complete the capping process^51^. The amino acid residues Pro^34^ and His^37^ of nsP1 are involved in the capping process and serve as the binding site for m^7^GMP, whereas the amino acids Asp^89^, Arg^92^, and Tyr^248^ play an important role in the methyltransferase activity^52^. The average predicted percent of intrinsic disorder (PPID) based on the outputs of four predictors in nsP1 protein (Fig. 4a) is 15.14% (Fig. 5a), which defines this protein as moderately disordered. The disordered regions lie within both N and C-terminal domain of nsP1, where the N-terminal region is required for capping process and C-terminal IDPR helps in the regulation of downstream translation.

nsP2 protein. This is the largest CHIKV protein that plays an important role in the pathogenic mechanism and in the life cycle of the virus^53^. In the viral replication, nsP2 display three key roles as helicase, triphosphatase, and protease^54^. nsP2 has two domains, N-terminal and C-terminal. The N-terminal domain shows NTPase-dependent helicase activity having helicase sequence motifs, such as motif 1 (residues 715–733), motif 1A (residues 738–752), motif 2 (residues 782–791), motif 3 (residues 811–818), motif 4 (residues 839–848), motif 5 (residues 908–929), and motif 6 (residues 940–954)^54^. The N-terminal domain is also associated with the NTPase-dependent RTPase activity to remove phosphate from the 59^th^ terminus of nascent RNA that acts as a substrate for capping reaction^54^. It also has nucleolar localization signal (NoLS region) and nuclear localization signal (NLS motif), positioned at residues 1005–1024 and 1182–1186, respectively^55^.

In other alphaviruses, nsP2 C-terminal domain (residues 1004–1327) is a papain-like protease domain, known as cysteine protease (thiol protease). However, a recent report revealed that in CHIKV, a cysteine residue can be catalytically replaced by serine. Hence, this study suggests that protease domain of CHIKV is not a papain-like protease^56^. It consists of two structural domains, such as a protease domain (residues 471–605) and a methyltransferase-like domain (residues 606–791). These two domains function as a single unit and are crucial for protease activity^57^. The functional mechanism of protease domain is related to deprotonation of a thiol group of cysteine residue from catalytic dyad at the active site (Cys^1013^ and His^1083^)^58,59^. The crystal structure of nsP2 protease domain of CHIKV have resolved (PDB ID: 3TRK) (Fig. 4bII). Although the analysis of the amino acid composition indicated that nsP2 has large net positive charge (+21), the disorder prediction revealed that the PPID value of the full-length nsP2 protein is only 0.50% (Fig. 5a). However, the helicase N-terminal ATP binding domain which plays a role in viral replication showing the PPID of 0.856% (Fig. 5b).

nsP3 protein. nsP3 protein constitutes the N-terminal macro domain, the Zn^2+^ binding domain, and the C-terminal variable tail domain. The crystal structure of macro domain (160 residues) has been resolved (PDB ID: 3GPG)^46^, where residues 30–37 represent a loop region and residues Thr^111^, Gly^112^, and Val^113^ form a binding site. The macro domain can interact with both mono and poly ADP-ribose and with RNA as well^60^. It has the ability to hydrolyze small substrate analog ADP-ribose-1-phosphate. A small Zn^2+^ binding domain follows macrodomain and contains four conserved cysteines (Cys^263^, Cys^265^, Cys^288^, and Cys^306^) where Cys^263^ and Cys^265^ are situated in the loop between the last two α-helices, crucial for replication^61^. Both macro domain and Zn^2+^ binding domain play a key role as regulators for the processing of non-structural polyprotein^61^. The third proline-rich C-tail domain is 205 residues long. It provides an insertion site for marker proteins. During the infection, components of stress granules G3BP1 and G3BP2 (host cells) directly bind to nsP3 protein to arrest RNA translation, and the interaction of G3BPs with nsP3 inhibits the stress granules formation^62^. Two short conserved repeat sequences near the C-terminal region of nsP3 interact with G3BPs. This interaction results in the depletion of G3BPs which leads to a reduction in CHIKV replication^63^. In disorder analysis, we found that the PPID value of this protein is 38.49% (Fig. 5a), and its C-terminal tail region shows the PPID value of 48.37% (Fig. 5b). Functional role of the C-terminal domain is not well known as of yet. However, it is assumed that it provides an insertion site for marker proteins. In other words, this analysis also showed the relevance of disordered regions of nsP3 for biological activities.

nsP4 protein. nsP4 acts as RNA-dependent RNA polymerase (RdRp) enzyme in CHIKV. It inhibits the phosphorylation at Ser^51^ residue of the α-subunit of elF2α, a eukaryotic translation initiation factor^64^. In addition to its polymerase activity, nsP4 also shows terminal adenylyltransferase activity, which is responsible for the synthesis of poly(A) tail in template-independent manner^65^. A recent report revealed that nsP4 is required for RNA synthesis assisted by other non-structural protein and possibly play a role in proper folding^65^. The N-terminal region (100 residues long) of nsP4 forms a partly unstructured domain, is necessary for the proper functioning of nsP4. This partly unstructured domain is followed by the catalytic domain with the established polymerase fold. A catalytic triad of GDD polymerase motif (residues Gly^464^, Asp^465^, and Asp^466^) is also important for polymerase activity^66^. The disorder analysis of this whole protein (Fig. 4d) gives the PPID value of 20.94% (Fig. 5a), but the N-terminal region (regulates polymerase activity) is characterized by the PPID of 76.00% (Fig. 5b). Therefore, one can target this region to stop its polymerase activity and thereby can inhibit virus replication within the host cell.

#### Analysis of the CHIKV structural proteins

**Figure 6:**
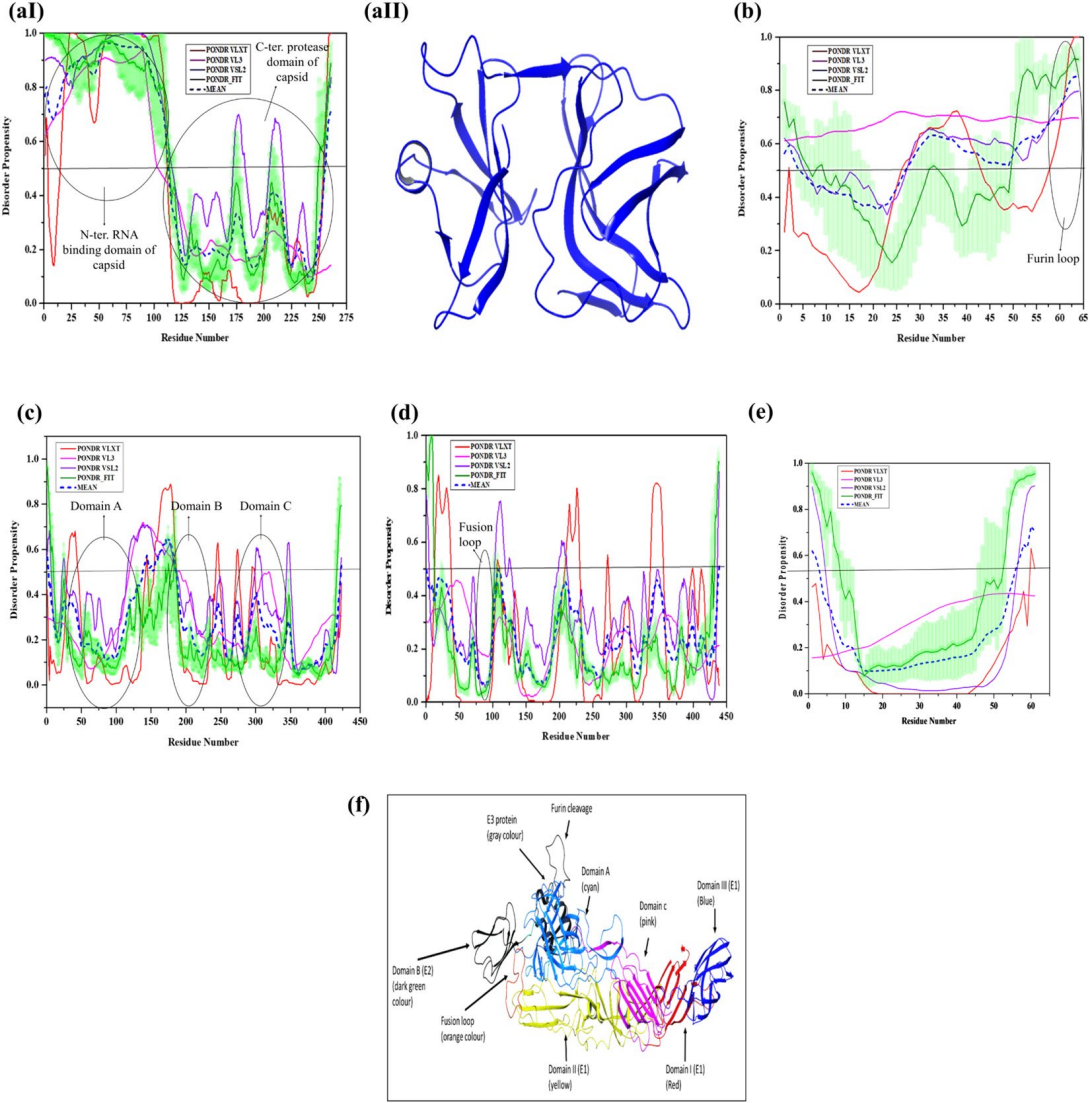
Diagrammatic representation of IDPs analysis of structural proteins of CHIKV. Plot represents the disorder analysis of (**aI**) capsid protein (residues 1–261, denoted as 1–261 at x-axis), shows complete disorderness in the N-terminal domain, black encircles represent domains (**aII**) the homology model of the C-terminal domain (blue color) of capsid protein (**b**) E3 protein (residues 262–325, denoted as 1–64 at x-axis), black encircle represents furin loop (**c**) E2 protein (residues 326–748, denoted as 1–423 at x-axis), black encircles represent domain A, B, and C (**d**) E1 protein (residues 810–1248, denoted as 1–439 at x-axis), black encircle represents fusion loop (**e**) 6K (residues 749–809, denoted as 1–61 at x-axis) protein (**f**) ribbon diagram of the crystal structure of p62-E1 heterodimer (PDB ID: 3N40); E1 domains I, II, and III are in red, yellow and blue colors respectively and fusion loop is in orange color; E2 domains A, B and C are in cyan, dark green and pink colors respectively; E3 protein is in grey color having furin loop (grey color) at the junction of E2 and E3 proteins.

Capsid protein. Capsid protein (CP) of alphavirus has an N-terminal RNA binding domain (residues 1–113) and the C-terminal protease domain (residues 114–261). The N-terminal domain constitutes two nuclear localization signal (NLS), residues 60–77 and 84–99. Although capsid protein is a cytoplasmic protein, it is assumed that its NLS may be involved in translocation of CP to the host nucleus^67^. The highly disordered N-terminal domain enriched with positively charged amino acid residues (Arg, and Lys) with high proline content. It has ribosome binding region (91–100 amino acid residues) that involved in protein-protein interaction. This domain also binds to the genomic RNA via 18 amino acid long coiled-coil α-helix^68^ and is required for the dimerization of capsid protein that negatively regulates host transcription^67^. This N-terminal domain is also involved in the interaction with RNA bound capsid proteins to form nucleocapsid core assembly in the cytoplasm of infected cells^67^. The nucleocapsids are then secreted out to the plasma membrane to interact with the C-terminal region of E2 for the initiation of virion budding. Residues 81–105 at the N- terminal domain of CP interact with RNA to form Capsid-RNA complex^69^. The C-terminal domain possesses a serine protease activity and is characterized by a chymotrypsin-like fold. This domain autoproteolytically cleaves CP from the nascent structural polyprotein. The report suggests that the C-terminal domain has numerous conserved amino acids, including a catalytic triad His^139^, Asp^161^, and Ser^213^, involved in the autoproteolytic activity that occurs at Trp^261^. Close to the substrate binding site of protease, a hydrophobic pocket is present, where binding of capsid to endodomain of the E2 glycoprotein occurs^70^. The predicted disordered analysis at the cleavage junction for CP is shown in Fig. 3d. IDP analysis of whole capsid protein (Fig. 6aI) shows PPID score of 45.59% (Fig. 5a). However, the PPID score of the N-terminal region comes out to be 100% (Fig. 5b). This N-terminal domain of CP is required for nucleocapsid core assembly that helps in virus budding.

p62 (pE2) protein. p62 precursor protein is a premature complex of E3-E2 protein. Processing and maturation of the glycosylated pE2 and E1 proteins takes place at the ER of infected host cells, where the polysaccharide chains are embedded on the surface of the envelope proteins to regulate cell receptor recognition, cell attachment, and entry into host membrane after viral fusion^71^. The envelope polyprotein encoded by CHIKV has four proteins arranged as E3-E2-6K-E1. Crystal structure of immature glycoprotein complex has been resolved (PDB ID: 3N40) (Fig. 6f). It forms a stable heterodimer with E1 protein to prevent premature cleavage of envelope polyprotein. After maturation, p62 is processed by host furin to E3 and E2-E1 heterodimer^72^ where we found disordered regions at E3-E2, E2-6K, 6K-E1 cleavage junction of envelope protein complex (Fig. 3e–g).

E3 protein. E3 is an α/β glycoprotein where β-hairpin is packed with three α-helices that forms a horseshoe-shaped structure^47^. E3 protein helps in the structural stability of E2 glycoprotein through modification of A and B domains in such a way that it can create a groove to accommodate fusion loop^47^. Another important function of E3 is to protect and stabilize the immature trimer (p62-E1) complex from acidic environment^73^. It also helps in low pH-mediated endocytosis of spikes (three E2-E1 heterodimers). Numerous polar residues are present at the N-terminal region (serve as E3 signal), required for the translocation and processing of p62 into ER lumen^74^. This signal plays a major role in trimer formation among E3-E2-E1^75^. The disorder analysis of E3 protein (Fig. 6b) gives PPID value of 65.62% (Fig. 5a), the highest score among all full-length CHIKV proteins. E3 glycoprotein has many disordered regions involved in stabilization of the E2-E3 complex structure and in the pH-mediated endocytosis of spikes. A furin loop present at the junction of E3 and E2 in the p62 precursor protein prevents premature cleavage of E1-p62 heterodimer^47^. This furin loop becomes disordered after the cleavage of the E1-p62 heterodimer.

E1 protein. Voss *et al*. reported the crystal structures of mature envelope glycoprotein complex (complex of E1, E2, and E3; PDB ID: 3N41, 3N42, 3N42, 3N43 and 3N44)^47^. The E1 protein is N-glycosylated at position N141^73^. It consists of three domains I, II, and III. The Domain II has a fusion loop (residues 893–910) at its tip, essential for the association of E1 and E2 glycoproteins. This fusion loop forms two H-bonds, first, between Ala^92^ of E1 and His^226^ of E2 (Domain B) and second, between Phe^87^ of E1 and His^29^ of (Domain A)^47^. The structure of CHIKV envelope is made up of spikes (three E2-E1 heterodimers). Eighty such spikes are present in a CHIKV virion, and those spikes mediate membrane fusion to deliver the viral genome into the host cell. The viral fusion is triggered by low pH (~5.5 to 6.0) dependent secretory pathway of endocytosis^73^. This is the reason why E1 fusion protein is expressed with E2 protein. However, disorder analysis of E1 protein (Fig. 6d) shows lower PPID value of 0.45% (Fig. 5a).

E2 protein. E2, a β-protein is crucial for cell receptor recognition, belongs to immunoglobulin superfamily consist of three domains (A, B, and C)^74^. Domain A and B of E2 protein forms a groove and this domain with unfolded fusion loop of E1 protein insert a β-hairpin into the groove to form a heterodimer complex. Further, these heterodimer forms spike in the envelope protein^47^. Domain B makes a contact with E3 protein via a long connector β ribbon. N-linked glycosylation of this protein occurs at Asn^263^ and Asn^273^ residues^73^. An acid sensitive region (ASR) (at 234^th^ residue) has been identified in E2 protein that triggers the initiation of conformational changes in the E1-E2 complex^47^. The disorder analysis of E2 protein (Fig. 6c) shows the PPID value of 12.29% (Fig. 5a) only. Though, these proteins do not show more disordered regions, however, domain B shows some disordered regions that are important for its functionality.

6K protein. 6K is a hydrophobic acylated protein involved in the envelope development and membrane permeabilization. The C-terminal domain of this protein mediates ER translocation of the E1 protein. 6K protein has the ability to increase membrane permeability by flippase of lipids from one side of the membrane to the other side that helps in virus budding^57^. The disorder analysis of this protein (Fig. 6e) gives the PPID score of 14.75% (Fig. 5a). Although the overall biological significance of IDPRs in this protein is not very clear, the disordered region of this protein plays an important role in virus budding.

CHIKV proteins play important role in the regulation of viral life cycle, pathogenic mechanisms, and immune evasion^1^. Our study revealed the abundant presence of disordered regions in structural and non-structural CHIKV proteins. A recent study evidenced the mutational changes happen in the E1 and E2 proteins that are identified as K211E and A226V (E1) and V264A (E2) polymorphism^7^.

## Concluding Remarks

It has been found that mutational changes occur in the disordered loop regions of E1 and E2 protein. These mutational changes trigger the conformational change in E1 protein to enhance the fitness of CHIKV propagating in Indian *Aedes agepty & Aedes albopictus*^7^. Fusion loop is disordered in nature that helps in dimer (E1 and E2 protein) formation. A recent study evidenced the mutational changes happen in the E1 and E2 proteins that are identified as K211E and A226V (E1) and V264A (E2) polymorphism^7^. These mutational changes are confined to the loop region of envelope protein, which is disordered in nature. They enhance the fitness of CHIKV residing in Indian *Aedes agepty & Aedes albopictus*. These mutations direct the conformational changes in the E1 protein. The current study suggests that IDPRs play a vital role in the structural flexibility and functional diversity of the CHIKV proteome and play diverse biological roles, such as cell cycle regulation, signaling, and protein stability of viral proteome. Therefore, we concluded that the disordered side of CHIKV proteome may provide a new angle to consider the pathogenic characteristic and virus-host interaction mechanism. In our study, we found that the N-terminal domain of capsid protein is completely disordered. This domain is required for the nucleocapsid assembly. Furthermore, the C-terminal domains of nsP3, E2 and E3 proteins have functional disordered regions. Since the role of IDPRs is well characterized in the regulation of life cycle of viruses, research has moved towards the development of the disorder based drug discovery strategies. The abundant disordered regions in CHIKV proteome could be an attractive target for drug designing. Since there is no vaccine/drug against chikungunya virus, a detailed analysis of CHIKV proteome is necessary to develop new drug by targeting the disordered regions.

## Methods

For disorder analysis, we used a reviewed polyprotein sequence of the S27-African CHIKV prototype, because this strain is a most causative strain of Chikungunya disease [UniProt ID: Q8JUX6 and Q8JUX5]. Previously, for disorder analysis, several specialized predictors have been developed, for example, PONDR^®^ pool [PONDR^®^ FIT^76^, PONDR^®^ VLS2^77^, PONDR^®^ VLXT^78^], as well as IUPred^79^, DisoPred^80^, DisEMBL^81^, GlobPlot^82^, SPRITZ^83^, and much more^84^. To evaluate the precision of disorder predictors, several aforementioned tools were compared within the frames of the Critical Assessment of Protein Structure Prediction (CASP)^85^. It was also indicated that because different predictors consider the occurrence of the intrinsic disorder in proteins from diverse perspectives, it is sagacious to use several computational tools to catch on the profusion of intrinsic disordered^17^. Typically, disorder predictors consider residues and regions as intrinsically disordered if their disorder score is above the 0.5 thresholds. The peculiarities of all predictors were considered while calculating the mean of all predictors.

PONDR^®^ VSL2^86^ gives one of the more accurate evaluations of disordered regions in a query protein, whereas PONDR^®^ VLXT is known as the most sensitive predictor for finding disordered based interaction sites^87^, and a meta-predictor PONDR-FIT as is more accurate than its individual component predictors, such as PONDR^®^ VLS2^77^, PONDR^®^ VLXT^78^, IUPred, FoldIndex, TopIDP. In our study, we have used PONDR^®^ FIT^76^, PONDR^®^ VSL2^77^, and PONDR^®^ VLXT^78^ for disordered analysis in polyprotein of CHIKV. Several reports suggested that IDPs/IDPRs play a central role in various molecular recognition events and in protein-protein interaction networks^30,88–92^. Some IDPs/IDPRs can undergo at least partial disorder-to-order transitions, when they get involved in interactions with specific binding partners that are needed for recognition, signaling, control, and regulation^93–95^.

## Electronic supplementary material

Supplementary Information
