# Supplementary material for: Deciphering the dark proteome of Chikungunya virus
Source: Sci Rep. 2018 Apr 11;8:5822. doi: 10.1038/s41598-018-23969-0 (PMC5895634; doi:10.1038/s41598-018-23969-0)
Supplement: Supplementary file 1 — Supplementary Information [file 41598_2018_23969_MOESM1_ESM.docx]

**Deciphering the dark proteome of Chikungunya virus**

Ankur Singh^1^, Ankur Kumar^1^, Rakhi Yadav^1^, Vladimir N. Uversky^2,3^, Rajanish Giri*^1,4^

^1^School of Basic Sciences, Indian Institute of Technology Mandi, Himachal Pradesh 175005, India. ^2^Department of Molecular Medicine and Byrd Alzheimer's Research Institute, Morsani College of Medicine, University of South Florida, Tampa, Florida, United States of America;^3^Laboratory of New Methods in Biology, Institute for Biological Instrumentation, Russian Academy of Sciences, Pushchino, Moscow Region, Russia. ^4^BioX Centre, Indian Institute of Technology Mandi, VPO Kamand, 175005, India.

*Email: [rajanishgiri@iitmandi.ac.in](mailto:rajanishgiri@iitmandi.ac.in)

Tel: +91-1905-267134

Fax: 01905-267138

**Running Title:** Dark proteome of Chikungunya virus

**Supplementary Table S1: Analysis of intrinsically disordered properties of CHIKV proteins**

| Protein | Length(MW: kDa) | pI | PPID _VLXT_ | PPID _VSL2_ | PPID _VL3_ | PPID _FIT_ | PPID _Mean_ |
| --- | --- | --- | --- | --- | --- | --- | --- |
| nsP1 | 535(59.89) | 6.62 | 33.08 | 25.23 | 18.32 | 12.14 | 15.14 |
| nsP2 | 798(89.47) | 9.09 | 12.66 | 8.40 | 0 | 2.63 | 0.50 |
| nsP3 | 530(58.08) | 4.91 | 48.49 | 49.62 | 42.64 | 27.73 | 38.49 |
| nsP4 | 611(68.24) | 6.96 | 24.55 | 27.82 | 17.51 | 8.83 | 20.94 |
| C | 261(29.61) | 10.29 | 41.38 | 56.32 | 39.46 | 48.27 | 45.59 |
| E1 | 439(47.46) | 6.37 | 17.31 | 8.88 | 0 | 4.78 | 0.45 |
| E2 | 423(47.05) | 8.37 | 15.37 | 22.70 | 17.49 | 5.2 | 12.29 |
| E3 | 64(7.34) | 6.49 | 40.62 | 65.62 | 100 | 40.62 | 65.62 |
| 6k | 61(6.70) | 6.75 | 3.28 | 16.39 | 0 | 27.86 | 14.75 |

**Supplementary Table S2: Analysis of intrinsically disordered properties of specific domains of CHIKV proteins**

| Protein name | Length(MW:kDa) | pI | PPID_VLXT_ | PPID_VSL2_ | PPID_VL3_ | PPID_FIT_ | PPID _Mean_ |
| --- | --- | --- | --- | --- | --- | --- | --- |
| nsP2  (N terminal) | 467(52.01) | 8.56 | 16.70 | 12.63 | 0 | 6.4 | 0.856 |
| nsP3  (C terminal) | 370(50.5) | 4.63 | 46.76 | 64.32 | 57.30 | 40.28 | 48.37 |
| nsP4  (N terminal) | 100(11.62) | 9.61 | 47 | 93 | 98 | 55 | 76.00 |
| C (N terminal) | 110 (13.23) | 12.08 | 84.55 | 100 | 100 | 100 | 100 |
